# Oxidative stress and hepatocarcinogenesis

**DOI:** 10.20517/2394-5079.2018.29

**Published:** 2018-08-01

**Authors:** Ying Fu, Fung-Lung Chung

**Affiliations:** 1 Laboratory of Molecular Biology, Center for Cancer Research, NCI, Bethesda, MD 20892, USA; 2 Lombardi Comprehensive Cancer Center, Georgetown University, Washington, DC 20007, USA

**Keywords:** Oxidative stress, DNA adduct, hepatocellular carcinoma, prevention, hepatocarcinogenesis

## Abstract

Hepatocellular carcinoma (HCC) is the second leading cause of cancer-related deaths worldwide. There are two major challenges for HCC, the first being that early detection is generally not applicable, and secondly, it is usually fatal within several months after diagnosis. HCC is an inflammation-induced cancer. It is known that chronic inflammation leads to oxidative/nitrosative stress and lipid peroxidation, generating excess oxidative stress, together with aldehydes which can react with DNA bases to form promutagenic DNA adducts. In this review, the evidence between oxidative stress and liver carcinogenesis is summarized. We focused on the potential of using DNA adducts as oxidative stress biomarkers for liver carcinogenesis.

## INTRODUCTION

Hepatocellular carcinoma (HCC) is the second leading cause of cancer-related deaths worldwide, because of late diagnosis and poor therapeutic outcome^[1–4]^. HCC accounts for 5.5% of all cancer cases globally, and particularly the incidence of HCC has been increasing in the US since the 1980s^[5,6]^. The incidence of HCC strongly correlates with liver inflammation from exposure to one or several risk factors including hepatitis B virus (HBV), hepatitis C virus (HCV), inherited metabolic diseases, heavy alcohol exposure, obesity, type 2 diabetes and aflatoxins^[7–13]^.

In this review, we will mainly discuss the role of oxidative stress in hepatocarcinogenesis. The search for reliable biomarkers for liver cancer has been executed in different areas: DNA methylation, genomics, proteomics, microRNA and liquid biopsy^[14–20]^. We want to highlight that promutagenic DNA adducts is a new field which need further investigations in the search of biomarkers for HCC.

## HEPATATOCARCINOGENESIS AND OXIDATIVE STRESS

More than 90% of HCCs arise in the context of hepatic inflammation^[21–29]^. Chronic liver inflammation leads to oxidative/nitrosative stress and lipid peroxidation (LPO), generating excess reactive oxygen species (ROS) and reactive nitrogen species (RNS), together with aldehydes which can react with DNA bases to form promutagenic DNA adducts through either endogenous or exogenous insults^[30]^. Oxidative stress has been demonstrated as an important factor to carcinogenesis since the first experiment on ROS-induced transformation of mouse fibroblast cells in the 1980s^[31]^. It has emerged as an important player in the development and progression of liver carcinogenesis for different etiologies (e.g., HBV- and HCV- induced liver diseases)^[32]^. HCC incidences in the USA are largely associated with HCV-related cirrhosis, but changes observed by epidemiological studies have attributed obesity and diabetes as risk factors as well^[33]^. The increased oxidative stress in obesity and diabetes may play a crucial role in hepatatocarcinogenesis^[34,35]^. Because oxidative stress drives genomic damage and genetic instability to cause mutations, and mutations play a crucial role in carcinogeneisis. This notion is supported by the chemopreventive effect demonstrated in a large number of epidemiology studies on the relationship of high fruit and, vegetable consumption with low cancer incidences, among which, antioxidants effects and maintenance of normal DNA repair capacity are indicated to be two crucial mechanisms of actions^[36,37]^. The same concept was illustrated when knocking out antioxidant defenses significantly increased the rate of liver cancer, e.g., knock-out mice lacking CuZuSOD (copperzinc superoxide dismutase) are found to increase liver carcinogenesis^[38]^. Another mouse model showed that knocking out nuclear respiratory factor-1 (Nrf1), an essential transcription for mediating oxidative stress, induces steatosis, fibrosis and liver cancer, eventually^[39]^.

The notion that oxidative stress induces HCC is also supported by studies on hemochromatosis. A positive correlation between mild/excess iron deposition and HCC in patients with hemochromatosis suggests a possible carcinogenic role for oxidative stress induced by iron through Fenton reactions^[40,41]^. In the iron-nitrilotriacetic acid rat model of hemochromatosis, elevated genotoxic products from oxidative stress, 4-hydroxyl-2-nonenal (HNE) and malondialdehyde (MDA), are found^[42]^. This increase is also accompanied by damaged cellular defense system, for instance, vitamin E level, GSH/GSSG ratio and superoxide dismutase are all decreased. HNE has the potential to damage genomic DNA and cause mutations, e.g., HNE adduct has been demonstrated to cause p53 mutations which are associated with more than 50% of HCC incidences^[43]^. A more important link was discovered in patients with hemochromatosis who suffered iron overload and p53 mutations following HCC development^[41,44–46]^; it suggests that oxidative stress is an underlying mechanism of HCC carcinogenesis^[44]^. The role of oxidative stress in liver carcinogenesis is also supported by the result of a multicenter study: using tissue microarray screening, cytochrome P450 1A2 (CYP1A2) oxidase in noncancerous tissue is found and validated as the only predictive factor for HCC recurrence^[47]^.

Oxidative stress is a crucial factor in the initiation and progression of HCC under various pathological conditions^[48]^. Oxidative stress can be induced by ROS produced in the mitochondria in non-alcoholic fatty liver disease, which damages hepatocytes, promotes pathologic polyploidization, triggers inflammation, and contributes to insulin resistance^[49–53]^. Additionally, oxidative stress is also involved in migration, invasion, and metastasis of HCC^[54–56]^. In that, biomarkers of oxidative stress can predict HCC risk and also the recurrence of HCC. Quantitative methods for the evaluation of oxidative stress can be divided into three categories: (1) determination of compounds modified by oxidative stress; (2) determination of the activity of antioxidant enzymes; and (3) determination of oxidative stress indicators containing transcription factors. Serum quantification of derivatives of reactive oxygen metabolites (d-ROM) level, a simple method for measuring hydrogen peroxide, is found to predict the risk of HCC recurrence after surgical resection or radiofrequency ablation (RFA)^[57]^. Since cancer is a genetic disease, we think that mutagenic DNA adducts that arise from oxidative stress have the potential to serve as more direct and precise biomarkers to predict HCC risk and recurrence. A major oxidative stress and promutagenic DNA adduct, 8-oxo-7, 8-dihydro-2’-deoxyguanosine (8-oxo-dG), was found to be increased during hepatocarcinognesis. It suggests a role of mutagenic DNA lesions in HCC formation^[58,59]^. In an HCV/HCC clinical trial, the result supports the hypothesis that HCV induces inflammation that causes oxidative DNA damage (increase of 8-oxo-dG, a DNA lesion), and promotes hepatocarcinogenesis.

LPO induced DNA adducts, including various propano- and etheno- adducts, have been investigated as potential lead markers for various types of inflammatory/oxidative stress cancer-prone diseases (e.g., chronic pancreatitis, Crohn`s disease, ulcerative colitis, alcohol related hepatitis, *H. pylori* infection) and cancer initiation/promotion^[60,61]^. It is also known that the propano DNA adducts [e.g., γ-hydroxy-1,*N*^2^-propanodeoxyguanosine (γ-OHPdG)] arisen from lipid peroxidation are mutagenic and associated with liver cancinogenesis^[62]^. The levels of propano DNA lesions are the balance of oxidative stress induced LPO and DNA repair. Nucleotide excision repair (NER) pathway is mainly responsible for repairing these bulky DNA adducts^[43,63,64]^. Patients with HBV may exhibit inefficiency of removing bulky DNA adducts because HBx protein has been shown to inhibit NER pathways through suppressing XPB and XPD helicases [transcription factor IIH (TFIIH)]^[65]^. We reason that DNA adducts possibly play a role of causing mutations by HBV, but further testing should be done to prove this hypothesis.

γ-OHPdG is an endogenous product of acrolein, a reactive aldehyde generated by LPO^[66]^. γ-OHPdG is known to cause G to T and G to A mutations that may involve critical genes such as *p53*^[67–70]^. Our recent studies demonstrated an association of the levels of γ-OHPdG with HCC development in a NER deficient mouse model with spontaneous HCC development. It is also found that antioxidants can suppress γ-OHPdG and prevent liver cancer significantly^[71,72]^. Further analysis found that GC>TA mutation is the dominant alteration, accounting for approximately 90% of mutations. The high GC>TA mutation frequency implies that γ-OHPdG may play a role in the mutagenesis of HCC development^[71,72]^. Understanding the role of DNA adducts of lipid peroxidation and the repair pathways involved may shed light onto mutagenesis during HCC development, and this knowledge will help us to find a way to its prevention^[73]^. To our knowledge, there is still no clinical data regarding LPO-derived DNA adducts as a predictive biomarker for HCC risk, we hope the ongoing interventional multi-center clinical trial “defined green tea catechin extract in preventing liver cancer in patients with cirrhosis (NCT03278925)” will shed some light on γ-OHPdG as a biomarker for liver carcinogenesis.

Thanks to recent advances in imaging modalities and the prevalence of a surveillance method for HCC, an increasing proportion of patients now receive local ablation therapy or curable resection. However, the high annual recurrence rate (approximately 20%) is still a huge hurdle before achieving long-term disease-free survival^[74]^. Neoadjuvant and adjuvant therapy for resectable HCC is still a difficult challenge. There are two major postoperative recurrence mechanisms: *de novo* carcinogenesis (usually late recurrence) and metastatic recurrence (usually occurs within one year and is related to intrahepatic metastasis)^[75]^. Precise prevention strategies are needed to target these mechanisms^[76]^. Three major strategies have been developed to address this issue^[77]^. The first one is a virus eradication method using interferon. But this method is not going to rescue the hepatocytes which have been damaged by hepatitis virus^[78]^. The second strategy is the use of anticancer drugs. Difficulties have been reported in the STROM trial (sorafenib as adjuvant treatment in the prevention of recurrence of hepatocellular carcinoma) and with the use of UFT (Tegafur-uracil)^[79]^. The last strategy is to induce differentiation of liver cancer cells. For example, using Pertinoin, an acyclic retinoid which can induce apoptosis and differentiation of cancer cells. This method has shown promising survival beneficial effects in a clinical phase II trial. Other than these strategies, branched chain amino-acid supplementation, vitamin K2 and acyclic retinoid have also been examined^[80]^. The reality is that no chemopreventive agent has been approved by FDA against HCC recurrence. There is still a lot of effort to be made to win this war against HCC recurrence. Future design may require focus on combination therapy. For instance, vitamin K2 and angiotensin-converting enzyme inhibitor have shown suppression effect on cumulative recurrence of HCC after curative therapy partially through reducing VEGF-mediated neovascularization^[81]^.

## FUTURE PERSPECTIVES

**Figure 1.**
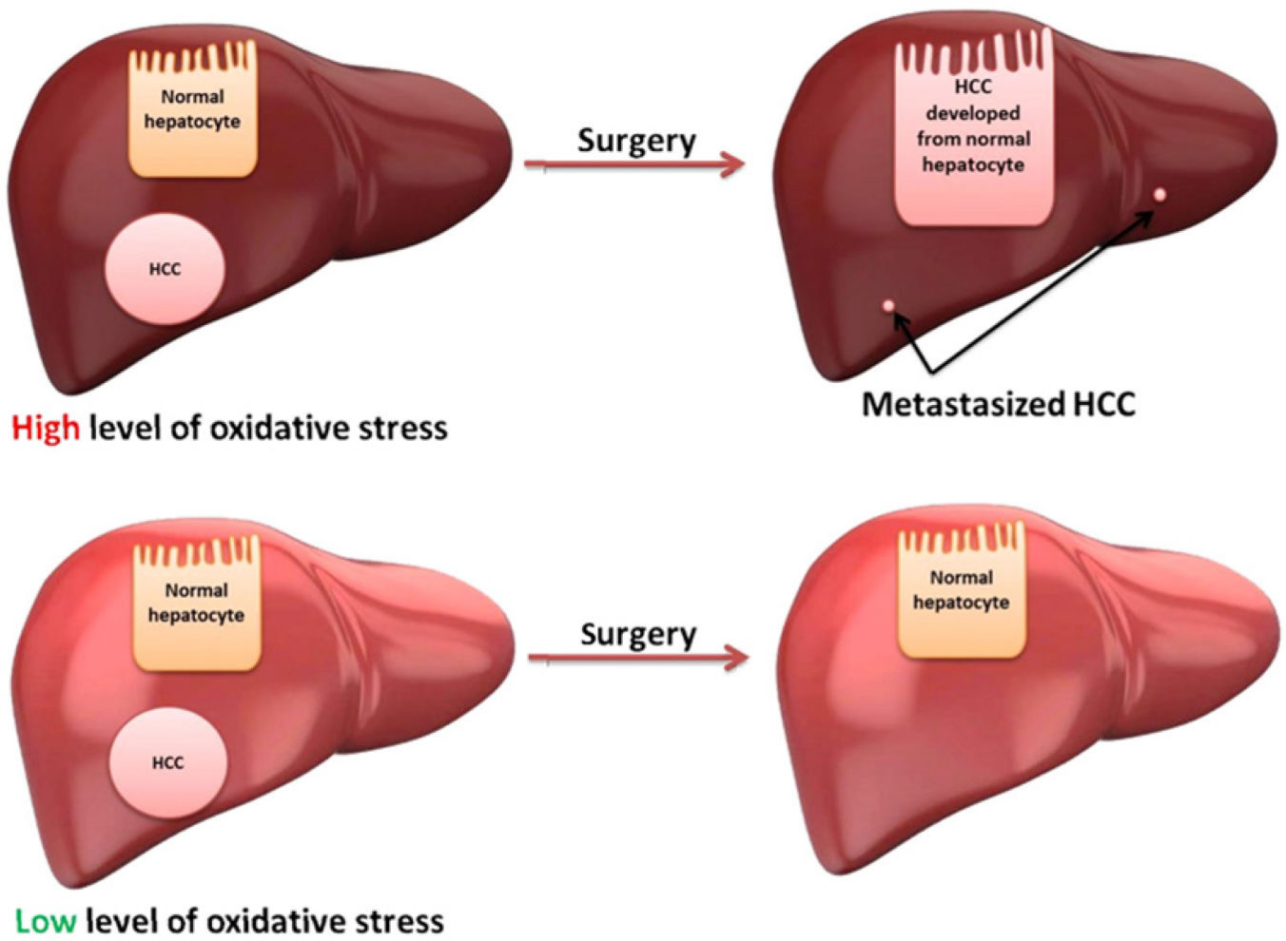
Oxidative stress and liver recurrence after surgery. HCC: hepatocellular carcinoma

Clinical trials using oxidative stress biomarkers for HCC and predicting HCC recurrence after curable surgery have been conducted [Figure 1]. Multi-center trials should be carried out to prove this application. The link between oxidative stress, DNA adducts, mutations, and cancer needs to be systematically studied; it is an area of study that can be accelerated by emerging technologies (e.g., next generation sequencing, Chip-seq, and SMART sequencing^[82]^). New technologies are needed to demonstrate in real-time link between exact DNA lesion sites (from normal tissue) and mutations (from tumor tissue). The idea of using antioxidants to prevent HCC recurrence has yet to be fully tested^[83–85]^. Use of oxidative stress markers to guide these trials warrants future investigation.
